# Optimization of extraction parameters of protein isolate from milk thistle seed: Physicochemical and functional characteristics

**DOI:** 10.1002/fsn3.4001

**Published:** 2024-03-13

**Authors:** Muhammed Ozgolet, Zeynep Hazal Tekin Cakmak, Fatih Bozkurt, Osman Sagdic, Salih Karasu

**Affiliations:** ^1^ Department of Food Engineering, Faculty of Chemical and Metallurgical Engineering Yildiz Technical University Istanbul Turkey

**Keywords:** emulsifying properties, foaming capacity and stability, milk thistle seed, optimization of protein extraction, protein isolate, response surface

## Abstract

In the current study, optimization of milk thistle protein extraction parameters was carried out in terms of purity and yield. In addition, the characterization of proteins isolated from milk thistle seeds was conducted. The optimal conditions for achieving the highest purity of protein (MTP) from milk thistle seeds were identified as extraction pH 9.47, temperature 30°C, and extraction time 180 min. Conversely, optimal values for overall protein yield (MTY) were determined at extraction pH 12, temperature 50°C, and extraction time 167 min. The proteins obtained under these two sets of conditions (MTP and MTY) demonstrated comparable oil absorption capacity (OAC), foaming, and emulsifying capabilities, as well as stability, aligning with findings from previous studies on seed protein. Both proteins had the highest protein solubilities at pH 11. Both proteins’ zeta potentials were closest to zero at pH 4, demonstrating their closeness to the isoelectric point. MTP and MTY had poorer antioxidant capabilities than the other protein isolates/concentrates. MTP and MTY contain high β sheet concentrations that might enhance thermal stability and lower the digestibility of proteins. In conclusion, the protein extraction process demonstrated a high potential for achieving both substantial yield and remarkable purity with some decent technological and functional properties, thus holding promise for various applications in diverse fields.

## Graphical abstract

In our study, optimization of milk thistle protein extraction parameters was carried out in terms of purity and yield. Therefore, milk thistle proteins have a high potential for thermal stability and low digestion.

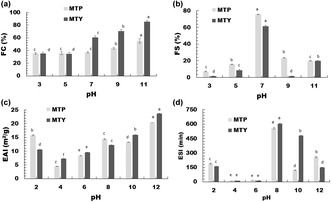

## 1 INTRODUCTION

Recently, food technologists have expressed interest in functional and enriched products with high nutritional quality since consumers are more aware of health‐improving benefits and more receptive to innovative food products. Moreover, the trends toward a sustainable food industry, considering environmental and economic concerns, have led to the revalorization of waste and by‐products as raw materials for other food products (Galali et al., 2020). For these reasons, by‐products obtained from oil‐processing have the potential to be utilized as valuable raw materials since they are rich in protein and fiber. Oilcakes and meals have been used to obtain protein isolates and hydrolysates (Choudhry et al., 2023; Mamone et al., 2019; Raikos et al., 2017). There are about 200 oil‐rich plants distributed worldwide (Kotecka‐Majchrzak et al., 2020). Even though soy, rapeseed, sunflower, coconut, and olive are the most abundant oils, in recent years, novel seeds such as milk thistle, chia, nigella, pumpkin, and hemp have been utilized for oil production. Awareness of the health benefits of oilseed and the oils obtained from oilseed has increased the global expansion of oil plant production (Kotecka‐Majchrzak et al., 2020). The edible oil cake is mostly used as feed for livestock due to its high amount of protein and other bioactive substances. However, the growing human population creates a need for a more logical use of by‐products from the food processing industry (Jozinović et al., 2017). Therefore, oilseeds and oils are increasingly common constituents in food products, and as a result, oil plant production is steadily increasing on a global scale (Usman et al., 2023).

*Silybum marianum* L. Gaertn, generally known as Mary thistle or milk thistle, is a widespread herbaceous plant in the family Asteraceae. Native to the Mediterranean, North Africa, and the Middle East, it is an annual or biennial plant. It can be cultivated or grow naturally. Besides, milk thistle is grown commercially for its exceptional therapeutic properties (Bijak, 2017). The proximate composition of milk thistle seed was 5.50% moisture, 6.30% ash, 6.46% crude fiber, 46.84% lipid, and 19.95% protein, while defatted seed contains 37.52% protein (Li et al., 2013). Previously published studies indicated that milk thistle seed polyphenols exhibit anti‐inflammatory, antioxidant, hypolipidemic, and anti‐carcinogenic activities (Khazaei et al., 2022; Mukhtar et al., 2023). Milk thistle seed oil can be considered as a natural source of vitamin E (Marceddu et al., 2022). In an ABTS• + assay, it was discovered that among eight different oilseed cakes, milk thistle flour made from a by‐product of cold pressing had the highest antioxidant activity. Total phenolic content (TPC) was also the highest in milk thistle oilcake (Bárta et al., 2021). The functional qualities of proteins isolated from milk thistle seeds were examined, and evidence demonstrated that proteins have the ability to take in fat and keep moisture, rendering them appropriate for utilization as additives in the production of acidic food items, including meat products, dairy alternatives, and protein‐rich beverages (Li et al., 2013). Thus, a study focusing on optimization of process parameters for protein extraction would be interesting. Response surface methodology has been utilized in different studies to optimize the extraction of proteins from various sources. However, there is no study in the literature optimizing extraction parameters for protein isolation from milk thistle seed.

A comprehensive investigation into the protein extraction and isolation from milk thistle seed has not been conducted so far. The primary aim of the present study was to enhance the extraction conditions for protein from milk thistle seeds through systematic control of key variables, including pH (ranging from 8 to 12), extraction temperature (from 30 to 60°C), and extraction duration (ranging from 1 to 3 h). Concurrently, an in‐depth exploration of the physicochemical and functional attributes of the resulting milk thistle seed protein concentrate (SPC) was conducted. The aim of this study was to enhance the efficiency of the extraction process while providing comprehensive insights into the characteristics of the obtained protein concentrate. To our knowledge, only one study focused on the functional properties of milk thistle seed, with an observation of a limited number of characteristics (Li et al., 2013). A commonly reported technique in the literature for the agri‐food industry is the extraction of proteins using an alkaline solution, which is then followed by isoelectric precipitation. The basic principle of this method is the significant solubility of proteins in alkaline environments and their limited solubility close to their isoelectric point. However, a number of variables, including pH, temperature, and extraction time, may impact protein solubility and, in turn, protein extractability. The protein extraction procedure from defatted milk thistle seeds has been optimized in this work using the response surface method to achieve the highest protein yield, and the purity of the protein isolate is assessed under the specific operational circumstances of pH, temperature, and extraction time.

## 2 MATERIALS AND METHODS

### 2.1 Materials

Cold‐pressed milk thistle (*Silybum marianum* L. Gaertn) oil by‐products were purchased from Tazemiz I. C. (Erdemli, Mersin). All chemicals were purchased from Sigma‐Aldrich (St. Louis, MO, USA) and of analytical grade.

### 2.2 Methods

#### 2.2.1 Preparation of milk thistle protein concentrate

Once the by‐product of milk thistle seeds arrived in our lab, it was kept at room temperature in sealed containers for further analysis. Milk thistle seed protein concentrate (SPC) was made from defatted seed flour according to the procedure proposed by Alu'Datt et al. (2012), with a slight modification. In short, the protein was extracted at a particular pH (pH 8–12) for the appropriate duration and temperature combinations (60–180 min, 30–50°C) using a sample to water ratio of 1:10 w/w. The aliquot was centrifuged at 12,000 × *g* for 10 min at 25°C. After the protein was precipitated at pH 4, it was centrifuged at 12,000 × *g* for 10 min at 25°C. The ideal pH of milk thistle seed protein precipitation was established prior to protein extraction. The dissolved proteins were precipitated and collected using centrifugation at 10,000 × *g* for 20 min at 4°C. They were then freeze‐dried and stored in sealed plastic bags for further analysis. The pH of the obtained supernatant had been adjusted to its isoelectric point with HCl.

#### 2.2.2 Optimization of milk thistle seed protein extraction conditions

The amount of protein for each extraction process was determined. For it, the Kjeldahl method was used to determine the nitrogen concentration of milk thistle protein concentrate (MTPC). The protein content of MTPC was calculated using the nitrogen‐to‐protein conversion factor of 6.25. Using a second‐order polynomial, data from 15 extractions were assigned to a response surface design using Minitab 18 software. Three independent variables were used at three levels in a Box–Behnken Design (BBD), as shown in Table 1. The factors pH (*x*_1_) from 8 to 12, temperature (*x*_2_) from 30 to 60°C, and extraction time (*x*_3_) from 1 to 3 h were studied. According to the BBD, a total of 15 trials were carried out in duplicate and randomly. Based on the highest protein yield and purity, optimum protein extraction conditions were determined. The protein yield and purity of milk thistle seed protein were chosen as the response variables individually. Finally, utilizing the optimum values of the factors, the protein isolate from milk thistle seed was obtained and employed in the subsequent analysis. Protein yield and purity were calculated using the following formulas (Equations 1 and 2):

(1) $$\text{Yield}\;\left(\%\right)=\frac{\text{Protein isolate obtained after freeze drying}\;\left(g\right)}{\text{Protein amount of the sample}\;\left(g\right)}\times 100$$

(2) $$\text{Purity}\;\left(\%\right)=\frac{\text{Protein amount of protein isolate}\;\left(g\right)}{\text{Protein isolate obtained after freeze drying}\;\left(g\right)}\times 100$$

**TABLE 1:** Independent variables and their levels in the response surface design.

Independent variables	Coded symbols	Coded factor levels		
		−1	0	1
pH	*x*_1_	8	10	12
Temperature (°C)	*x*_2_	30	40	50
Extraction time (min)	*x*_3_	60	120	180

#### 2.2.3 Oil absorption capacity

500 μL of corn oil and 50 mg of protein were mixed in an Eppendorf tube. Following the centrifugation (5000 *g*, 5 min) of the mixture, the filtrate was removed and the tubes were weighed. The oil absorption capacity was determined using Equation 3 (grams of absorbed oil per gram of protein isolate).

(3) $$\mathrm{OAC}\;\left(g/g\right)=\frac{W_{2}-W_{1}}{W_{0}}$$

let *W*_0_ represent the weight of the dry sample in grams, *W*_1_ represent the combined weight of the tube and dry sample in grams, and *W*_2_ represent the weight of the sediment and tube in grams. The analyses were conducted in duplicate (Tomotake et al., 2002).

#### 2.2.4 Emulsifying properties

The stability of the protein and the emulsion activity were assessed using a turbidimetric technique described by Bozkurt et al. (2021). A homogenizer (IKA Ultra Turrax T25, Schott Iberica, SA, Spain) was used to blend 10 mL of sunflower oil with 30 mL of a 1% protein solution for 90 s at a speed of 10,000 rpm. The sample volume was reduced to 50 μL, and 5 mL of a 0.1% sodium dodecyl sulfate (SDS) solution was added. Using a UV spectrophotometer, the diluted solution's absorbance was determined at 500 nm (Shimadzu UV 1800; Kyoto, Japan).

The formulas utilized to measure the emulsion activity index (EAI) and emulsion stability index (ESI) are as follows (Equations 4 and 5):

(4) $$T=2.303\times \frac{A}{l}$$

where *T*: turbidity, *A*: absorbance of hydrolysates at 500 nm, and *l*: the light path of a cuvette (m)

(5) $$\mathrm{EAI}=2\cdot T\cdot \frac{r}{c\cdot V}$$

The variables in question are as follows: *r* represents the dilution factor, *c* represents the weight of protein per unit volume in grams per milliliter (g/mL), and *V* represents the volume percent.

In order to assess the stability of the emulsion, the hydrolysate emulsions were placed in storage at a temperature of 4°C for a duration of 10 min. The emulsion stability index (ESI) is the quantitative measure of the difference between 0 and 10 min. The emulsion stability was determined by applying the following formulas (Equation 6).

(6) $$\mathrm{ESI}=\frac{T∆t}{∆T}$$

where *T*: initial turbidity value, Δ*T*: change in turbidity after 10 min, and Δ*t*: time interval between two measurements (min).

#### 2.2.5 Protein solubility

Protein solubility was determined using the procedure suggested by Shevkani et al. (2015) with a minor modification. In brief, 200 mg of each protein sample was dispersed in 20 mL of deionized water, and 1 or 0.1 N HCl and 1 or 0.1 N NaOH were used to adjust the pH of the mixture to 3, 5, 7, 9, and 11. Prior to being centrifuged at 7500 *g* for 15 min, the mixture was agitated for 30 min at a temperature of 20°C adjusted by a magnetic stirrer. The protein concentration in the supernatant was measured using the Kjeldahl method, employing a nitrogen‐to‐protein conversion factor of 6.25. The solubility of proteins was then calculated as follows (Equation 7):

(7) $$\text{Solubility}\;\left(\%\right)=\frac{\text{Protein content of supernatant}}{\text{Total protein content of the sample}\;}\times 100$$

#### 2.2.6 Foaming capacity and foaming stability

The procedure proposed by Garg et al. (2020) was utilized to measure foaming stability (FS) and foaming capacity (FC). Each protein sample, weighing 2 g, was evenly distributed in 100 mL of deionized water. The pH of the solution was then changed to 2, 4, 6, 8, 10, and 12 using either 1 or 0.1 N HCl and 1 or 0.1 N NaOH. The solution was then vigorously mixed for a duration of 2 min. Foaming capacity and stability were calculated using the following formula (Equations 8 and 9):

(8) $$\text{Foaming capacity}\;\left(\%\right)=\frac{{\text{Volume}}_{\text{after whipping}}-{\text{Volume}}_{\text{before whipping}}}{{\text{Volume}}_{\text{before whipping}}}\times 100$$

(9) $$\text{Foaming stability}\;\left(\%\right)=\frac{\text{Foaming volume after}\;30\;\min}{\text{Initial foam volume}}\times 100$$

#### 2.2.7 Antioxidant activity

The antioxidant potentials of MTP and MTY were assessed using the 1,1‐diphenyl‐2‐picrylhydrazyl (DPPH) and ferric‐reducing antioxidant power (FRAP) methods, respectively. In the DPPH method, 0.1 mL of MTP and MTP at various concentrations were mixed with 4.9 mL of DPPH solution (4.0 mg/100 mL methanol). The DPPH and sample extract mixture was kept at a temperature of 20°C for 20 min. The DPPH and sample extract mixture was maintained at a temperature of 20°C for a duration of 20 min. Finally, the absorbance at 517 nm was measured to determine the concentration causing an inhibition of 50% of the DPPH radical (Kayacan et al., 2022). The FRAP assay was performed in accordance with Bozkurt et al. (2021). The FRAP reagent was produced by combining 25 mL of 300 mM acetate buffer (pH 3.6), 2.5 mL of 10 mM TPTZ solution in 40 mM HCl, and 2.5 mL of 20 mM FeCl_3_‐6H_2_O. 15 mg of MTP and MTY were dissolved in 1 mL of phosphate buffered saline (PBS). 100 μL of MTP and MTY were added to the 900 μL of PBS solution. Two milliliters of a new FRAP reagent were added to the mixture. The solution was subsequently placed in a water bath at 37°C for 15 min. At 593 nm, each sample's absorbance was measured. Trolox equivalents in mg gallic acid/g sample were used to express the results.

#### 2.2.8 Zeta potential

The zeta potential was measured in accordance with the procedure proposed by Liu et al. (2019). In brief, 20 mg of samples were dissolved in 10 mL of PBS at pH 9. Zeta potentials were measured at each number as the pH was incrementally adjusted (8.0, 7.0 … 4.0) using 1 M HCl. A Zetasizer Nano‐ZSP instrument (Malvern Instruments, Worcestershire, and the U.K) was used to measure the overall surface charges of the samples.

#### 2.2.9 FTIR

FTIR analysis of milk thistle protein was collected at a resolution of 4 cm^−1^ in the infrared wavelength range of 4000–650 cm^−1^, with 16 scans per spectrum. An FTIR (Bruker Tensor 27, Germany) spectrometer with an attenuated total reflectance accessory was used for all measurements. The OPUS software (Bruker Optics, Germany) was used to acquire spectra, normalize them, and subtract the reference spectrum. To ascertain the secondary structures of the protein, deconvolution was employed on all spectra within the amide I (1600–1700 cm^−1^) area using Origin 2023 (Originlab Corporation, MA, USA) software.

#### 2.2.10 SDS‐PAGE analysis

The SDS‐PAGE analysis was carried out in accordance with Laemmli's method (Laemmli, 1970). Milk thistle protein extract was dissolved in distilled water at a concentration of 10 mg/mL. The extract solution was mixed with sample buffer (0.5 M Tris–HCl pH 6.8, glycerol, 10% SDS, w/v, 0.5% bromophenol blue, β mercaptoethanol) at a ratio of 1/1 (v/v). It was then heated at 95°C for 3 min. The electrophoresis tank was filled with electrolyte buffer, including 25 Mm Tris, 0.1% SDS, and 192 mM glycine. 10 μL of sample was loaded onto 4% stacking gel (w/v) and 7.5–30% gradient polyacrylamide gel (w/v) with the help of a microsyringe. The gel was electrophoresed vertically using a Mini‐PROTEAN®System (Bio‐Rad Laboratories, Inc, USA) at a constant current of 20 mA. The gel was subsequently dyed with a solution containing 0.25% (w/v) Coomassie Brilliant Blue is a type of dye. A mixture of water/methanol/acetic acid (6/3/1, v/v/v) was used in the dye removal process. The gel was photographed with an imaging system (Biorad Gel Doc EZ, Bio‐Rad Laboratories Inc, USA).

#### 2.2.11 Statistical analysis

All treatments were carried out in triplicate, and the corresponding mean value is shown with the standard deviation. RSM using Minitab 18 was performed to optimize the protein extraction. The statistical analysis was performed with SPSS software (version 16; SPSS Inc., Chicago, IL, USA). Tukey's multiple range test was employed as the multiple comparison test for evaluating the statistical comparison between means at a significance level of 0.05. The STATISTICA program (version 12; Statsoft, Tulsa, OK, USA) was used to calculate the rheological parameters of the fitted models and the goodness of fit, measured by the coefficients of determination (*R* ^2^). The curves were plotted using a Microsoft Excel spreadsheet (version 2016; Microsoft Office, Redmond, WA, USA).

## 3 RESULTS AND DISCUSSION

### 3.1 Response surface optimization

RSM was utilized to evaluate the impact of various process parameters on the protein yield and purity of milk thistle protein isolate. In accordance with the BBD, a total of 15 experiments were conducted in triplicate and in a random order (Table 2). Based on maximum yield and purity, optimum pH, temperature, and extraction time values for the extraction process were determined. The maximum yield was obtained at pH 12 and 50°C for 168 min, whereas the maximum purity was provided at pH 9.47 and 30°C for 180 min. In Table 3, the estimated effects of the independent variables at the linear and quadratic levels and their interactive relationships with the dependent variables are displayed. The *p*‐value, coefficient of determination, and lack of fit were used to evaluate the suitability of the suggested model. The models of protein yield and purity are significant at *p* < .05, and the model was validated by higher values of the coefficient of determination (*R* ^2^ > .96). For all of the fitted models, the lack of fit was determined to be insignificant (*p* > .05). These findings show that the proposed models accurately reflect the data for all the response variables. The following were the yield and purity quadratic regression models in coded units:

$$\begin{array}{c} \text{Purity}\;\left(\%\right)=77.73–2.705\;x_{1}+0.362\;x_{2}+0.735\;x_{3}–3.914\;{x_{1}}^{*}\;x_{1}\\ +2.511\;{x_{2}}^{*}\;x_{2}–0.549\;{x_{3}}^{*}\;x_{3}+1.112\;{x_{1}}^{*}\;x_{2}+1.788\;{x_{1}}^{*}\;x_{3}–2.332\;{x_{2}}^{*}\;x_{3} \end{array}$$

$$\begin{array}{c} \text{Yield}\;\left(\%\right)=45.1–7.8\;x_{1}–0.27\;x_{2}–0.100\;x_{3}+0.649\;{x_{1}}^{*}\;x_{1}\\ +0.0036\;{x_{2}}^{*}\;x_{2}–0.000178\;{x_{3}}^{*}\;x_{3}–0.0077\;{x_{1}}^{*}\;x_{2}\\ +0.0045\;{x_{1}}^{*}\;x_{3}+0.00211\;{x_{2}}^{*}\;x_{3} \end{array}$$

where, *x*_1_ = pH, *x*_2_ = extraction temperature (°C), *x*_3_ = extraction time (min).

**TABLE 2:** Box–Behnken design (BBD) and responses for the optimization of the protein concentrate extraction of defatted milk thistle seed.

Run	pH	Temperature (°C)	Time (min)	Purity (%)	Yield (%)
1	8 (−1)	30 (−1)	120 (0)	79.14 ± 0.10^c^	16.05 ± 0.07^m^
2	8 (−1)	40 (0)	60 (−1)	77.67 ± 0.05^f^	24.20 ± 0.06^i^
3	8 (−1)	40 (0)	180 (1)	74.36 ± 0.06^j^	17.50 ± 0.05^k^
4	8 (−1)	50 (1)	120 (0)	78.84 ± 0.10^d^	15.82 ± 0.07^m^
5	10 (0)	30 (−1)	60 (−1)	76.26 ± 0.03^h^	24.13 ± 0.09^i^j
6	10 (0)	30 (−1)	180 (1)	83.60 ± 0.08^a^	17.08 ± 0.07^L^
7	10 (0)	40 (0)	120 (0)	77.33 ± 0.12^g^	23.93 ± 0.05^j^
8	10 (0)	40 (0)	120 (0)	78.53 ± 0.10^e^	26.18 ± 0.07^g^
9	10 (0)	40 (0)	120 (0)	77.45 ± 0.06^fg^	24.98 ± 0.08^h^
10	10 (0)	50 (1)	60 (−1)	80.45 ± 0.07^b^	29.89 ± 0.12^e^
11	10 (0)	50 (1)	180 (1)	78.46 ± 0.08^e^	27.89 ± 0.10^f^
12	12 (1)	30 (−1)	120 (0)	71.59 ± 0.06^L^	46.46 ± 0.04^a^
13	12 (1)	40 (0)	60 (−1)	68.60 ± 0.09^m^	35.39 ± 0.09^d^
14	12 (1)	40 (0)	180 (1)	72.44 ± 0.07^k^	37.85 ± 0.10^c^
15	12 (1)	50 (1)	120 (0)	75.74 ± 0.08^i^	39.61 ± 0.06^b^

*Note:* The different lowercase letters in a column represent statistical significance (*p* < .05).

**TABLE 3:** Estimated coefficients of the fitted second‐order polynomial model, representing the relationship between the responses and the independent variables.

	Purity (%)		Yield (%)	
	Coefficient	*p* value	Coefficient	*p* value
Constant	77.73	.000	25.030	.000
*x*_1_ (pH)	−2.705	.001	10.843	.000
*x*_2_ (Temperature)	0.362	.418	1.938	.205
*x*_3_ (Time)	0.735	.134	−0.785	.580
*x*_1_ *·x*_2_	1.112	.114	−0.155	.937
*x*_1_ *·x*_3_	1.788	.028	0.540	.785
*x*_2_ *·x*_3_	−2.332	.010	1.265	.531
*x*_1_ *·x*_1_	−3.914	.001	2.595	.242
*x*_2_ *·x*_2_	2.511	.009	0.360	.861
*x*_3_ *·x*_3_	−0.549	.406	−0.640	.757
*R* ^2^	96.57		93.47	
*R* ^2^‐adj	90.39		81.72	
Lack‐of‐fit		.205		.053
Model		.004		.017

### 3.2 Effect of process parameters on protein yield of protein isolates

The yields of protein isolate ranged from 15.82 to 46.46%. The pH had a significant impact on yield at the linear level (*p* < .05), according to the analysis of variance. From Table 3, the coefficients for linear terms *x*_1_, *x*_2_, and *x*_3_ show that pH has the highest effect on the yield, followed by temperature and time, respectively. With increases in pH levels at a linear level, a considerable rise in protein isolate production was observed. It may be attributed to the fact that the negative charge of proteins increases as the pH rises and the electrostatic repulsion between them increases. As a result of these protein–water interactions, protein solubility increases (Lestari et al., 2010). With raising pH from 6.25 to 10, during longer extraction times, there was an increase in the yield of protein isolates from grass peas (Feyzi et al., 2018). The protein yield increased with an increase in temperature, while it decreased with an increase in extraction time. However, the linear and quadratic effects of temperature and extraction time were insignificant (*p >* .05). Protein yield decreased to 24.20% from 17.50% at a constant pH and temperature (pH 8 and 40°C). In addition, at constant temperature of 40°C and an extraction time of 60 min, the increase in pH from 8 to 12 led to an increase in yield from 24.20% to 35.39%. Protein yield was found to be maximum (46.46%) at 12 pH, an extraction temperature of 30°C, and an extraction time of 120 min, whereas the lowest protein yield (15.82%) was observed at 8 pH, 50°C, and a 120 min extraction time. The low protein content at high temperatures may be related to the high dissolubility of starch at higher temperatures (Vaidya et al., 2017). In the case of interaction terms, positive coefficients reflect the effects in a synergistic manner. The coefficients of terms *x*_1_ *x*_3_ and *x*_2_ *x*_3_ showed the impact on protein yield synergistically, while the term *x*_1_ *x*_2_ manipulated the protein yield in an antagonistic manner. However, the interaction between the parameters was found to be insignificant at *p* > .05.

### 3.3 Effect of process parameters on the purity of protein isolates

The purity of protein isolates ranged from 68.60% to 83.60% under the specified experimental conditions (Table 2). The combination of process conditions at pH 10, an extraction temperature of 30°C, and an extraction time of 180 min resulted in the observation of the highest purity of protein isolates. The effect of pH on purity at the linear and quadratic levels was significant (*p* < .05). The negative coefficient of pH reflects that an increase in pH causes a decrease in purity. In addition, the negative coefficient at the quadratic level for pH indicates maximum purity near the central point, which decreases with an increase in pH. Alkali pH causes easier interaction between proteins and the solvent, thus promoting higher protein solubility. Beyond pH 10, protein molecules unfold or denature due to a high alkaline medium, which results in a distorted conformation with modified hydrophobicity (Garg et al., 2020). In addition, higher pH causes easier starch extraction due to higher starch swelling, thus diluting protein concentration (Hoang, 2012). The positive coefficient of temperature at the quadratic level shows a minimum response at the center, which increases with distance from the central point. From the coefficients of interaction terms, pH displayed a synergistic effect with extraction time on protein purity. The pH value at the center point and the maximum extraction time led to the highest protein purity (83.60%). Temperature influenced protein purity and extraction time in an antagonistic manner. Therefore, at lower temperatures and high extraction times, protein purity increases. In the study, protein extraction was enhanced with increased extraction times due to the longer duration for the diffusion of proteins out of the seeds (Koocheki et al., 2009). The interaction between pH and temperature was found to be insignificant (*p* > .05).

### 3.4 Process parameter optimization and validation

The optimal conditions were determined using a numerical optimization process that maximized protein yield and protein purity while keeping independent variables within the range. According to the statistical analysis, the optimal conditions were 9.47 pH, 30°C extraction temperature, and 180 min extraction time for protein purity, and 12 pH, 50°C extraction temperature, and 167 min extraction time. When the optimum values of the independent variables were entered into the regression equation, 41.01% protein yield and 82.66% purity were obtained. To test the model equation's suitability for predicting optimum protein yield and purity, three independent real experiments were performed at optimal conditions. Experiment validation of optimized conditions revealed 40.18% protein yield and 84.95% protein purity. The gap between the experimental and predicted values of protein yield and purity under optimized conditions were less than 3%, demonstrating the model's adequacy. From now on, the protein isolates obtained at optimum conditions for the highest protein yield and purity will be referred to as MTY and MTP respectively.

### 3.5 Functional and technological properties of protein isolates at optimum points

Functional and technological properties of protein isolates are important to improve food product quality. The protein isolate obtained at the points with the highest protein yield and purity were examined. In Table 4, the various functional properties of milk thistle seed protein isolate prepared under optimal conditions were displayed.

**TABLE 4:** Certain functional and technological characteristics of MTP and MTY.

Physical parameters	MTP	MTY
Protein (%)	84.95 ± 0.28	75.54 ± 0.19
IC_50_ (μg/mL)	6.03 ± 0.05	5.16 ± 0.07
FRAP (μg gallic acid/g sample)	166.33 ± 0.14	180.00 ± 2.70
OHC	2.06 ± 0.04	4.67 ± 0.02
Protein solubility		
pH 3	15.46 ± 0.39^d^	18.54 ± 0.11^e^
pH 5	7.21 ± 0.05^e^	30.13 ± 0.11^d^
pH 7	18.14 ± 0.12^c^	36.74 ± 0.17^c^
pH 9	66.99 ± 0.94^b^	78.81 ± 0.12^b^
pH 11	87.60 ± 0.10^a^	99.67 ± 0.91^a^
Zeta potential		
pH 4	−15.7 ± 0.3^a^	−20.6 ± 0.7^a^
pH 5	−26.4 ± 0.5^b^	−24.5 ± 1.1^b^
pH 6	−26.0 ± 0.6^b^	−25.6 ± 0.3^b^
pH 7	−26.6 ± 0.6^b^	−27.7 ± 0.3^c^
pH 8	−27.4 ± 0.5^b^	−33.8 ± 0.7^d^
pH 9	−27.2 ± 0.6^b^	−34.3 ± 0.5^d^

*Note:* The different lowercase letters in a column represent statistical significance (*p* < .05).

#### 3.5.1 Oil absorption capacity

Oil absorption capacity (OAC) demonstrates proteins’ ability to interact with lipids, which is a critical functional property in the food industry. The amount of oil retained by the protein or peptide is also defined as OAC. The oil absorption capacities of MTY and MTP were 4.67 and 2.06, respectively. The OAC of MTP is comparable to the OAC of soy protein isolate (2.61), buckwheat protein (2.81), and hemp seed protein (1.79) and higher than the OAC of casein 0.88 (Tang et al., 2006; Tomotake et al., 2002). The OAC values of two different black cumin seed protein isolates were found to be 6.56 and 8.27 (Trigui et al., 2021). OAC of Indian black gram protein isolates was observed at 5.5–6.3 g/g (Wani et al., 2015). The protein isolates of the residues from perilla seed following different oil extraction procedures, such as solvent‐extraction, hot‐pressing, and cold‐pressing, offer an OAC of 2.2–2.5 g/g (Zhao et al., 2021). The OAC values of different protein isolates offer a wide range of OAC values. The OAC of a protein was determined by several factors, including protein type, its surface hydrophobicity, lipophilic groups, conformational properties, and non‐protein compounds in the protein isolate (Pham et al., 2017). MTY contains a higher non‐protein fraction, which might cause higher oil absorption. Similarly, in different studies, watermelon seed flour (35.66% protein) and watermelon seed protein isolate fractions (>84% purity) were examined for their oil absorption capacities (El‐Adawy & Taha, 2001; Wani et al., 2011). OAC of seed flour was found to be higher than OAC of protein isolate fractions; offering non‐protein fraction in watermelon seed influenced OAC favorably.

#### 3.5.2 Protein solubility

Protein solubility is of utmost importance due to its effect on functional characteristics such as emulsification, whipping, foaming, etc. Protein solubilities (PS) of MTP and MTY depending upon pH were presented in Table 4. The minimum PS of MTP and MTY were at pH 5 and pH 3, which is thought to represent closeness to the isoelectric point, respectively, and the highest protein solubility was observed at pH 11 for both MTP and MTY. After pH exceeded 6, a rapid increase was observed in PS. Protein isolates obtained from lupin, quinoa, and chia were least soluble at pHs of 5, 6, and 4, respectively (Mäkinen et al., 2015; Schlegel et al., 2019; Vázquez‐Ovando et al., 2013). Melon seed protein concentrate showed the lowest solubility at pH 4.5, which is close to the isoelectric point of the protein, and PS demonstrated an increasing trend with increasing pH up to 12 (Ogunbusola et al., 2022). The wampee seed protein and soy protein isolates have the lowest PS at pH 3 and 4.5, respectively, and the solubility increased with the rise in pH (Liu et al., 2018). A similar trend was noticed in hemp seed protein, whose PS increased from 0% to around 80% following isoelectric point pH 5 up to pH 10 (Hadnađev et al., 2018). The results of the studies mentioned above are comparable to ours. It is due to the fact that the solubility of proteins is often lowest at isoelectric pH or close to it since there are no net charges accessible on the protein surface. The protein solubility is improved by increasing net negative or positive surface charge on the protein by being away from the isoelectric point.

#### 3.5.3 Emulsifying characteristics

**FIGURE 1:**
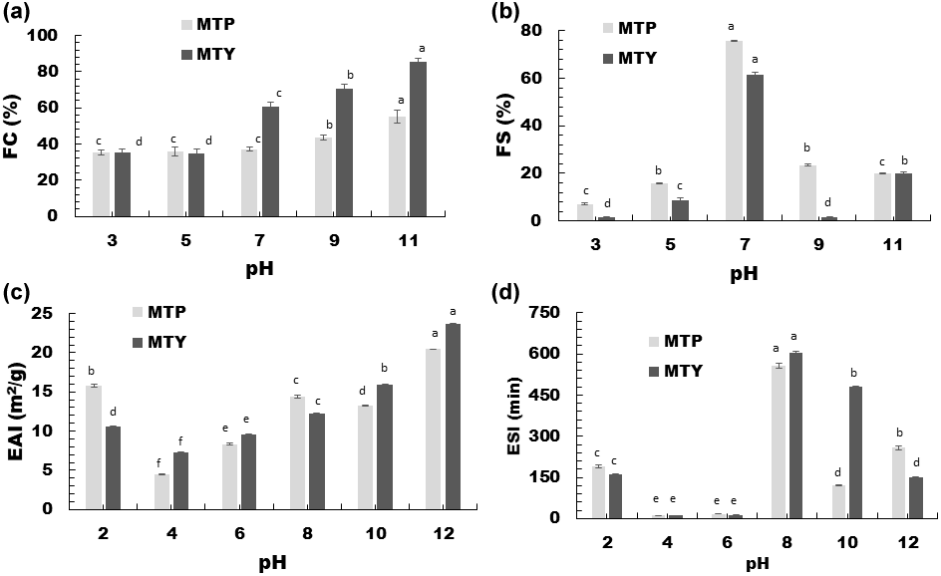
(a) Foaming capacity, (b) foaming stability, (c) emulsion activity index, and (d) emulsion stability index of MTP and MTY proteins. Values are mean ± SD. The different lowercase letters in the columns indicate statistical significance between the values of MTP and MTY proteins at each pH level (Tukey's test, *p* < .05).

Food proteins are amphiphilic molecules that can be absorbed at the emulsion interface and stabilize the oil droplets. The position and number of amino acids in the polypeptide chain primarily determine the functional properties of such proteins. A good emulsifier must have an optimal balance of polar and non‐polar groups in order to have adequate water solubility and surface activity (Padial‐Domínguez et al., 2020). As a result, changes in the pH of the environment influence the net charge of the protein as well as its conformation at the interface, affecting their emulsifying capacities and stabilities. In this study, the EAI and ESI of MTY and MTP were determined at a variable pH from 2 to 12 in order to demonstrate the impact of pH. The emulsion activity index (EAI) and emulsion stability index (ESI) values are commonly used to express emulsification capacity. EAI correlates a protein's capacity to coat an interface by determining the interface area stabilized per unit weight of protein (m^2^/g). The EAI and ESI values of MTY and MTP at different pH values are shown in Figure 1. EAI values ranged from 7.30 to 23.64 m^2^/g for MTY and 4.48 to 20.45 m^2^/g for MTP, whereas ESI values of MTY and MTP were in the range of 10.26–605.46 min and 10.48–557.14 min, respectively. Both EAI and ESI values showed an increasing trend as the distance from the isoelectric distance increased. The EAI value of acid‐precipitated soy protein concentrate (86.7% protein) was 7.27 m^2^/g (Rao et al., 2002). In addition, eight mung bean protein isolates showed EAI of 6.735–8.598 m^2^/g (Sun et al., 2022). ESI of hemp seed proteins was found to be around 50 min (Li et al., 2022). In another study, peanut protein isolates had an ESI of around 90 min at pH 9 (Zhang & Lu, 2015). Six different pea protein isolates had EAI values of 9.27–291.94 m^2^/g, with the highest EAI found at the highest pH studied (pH 8). In the same study, the highest ESI (550 min) was observed at pH 8, while the lowest ESI (1.5 min) was found at pH 5 (Barac et al., 2010). EAI and ESI of Persian lime seed protein isolate were minimum at pH 4, and EAI and ESI values increased with distance from the isoelectric point (Fathollahy et al., 2021). Six different pea protein isolates had EAI values of 9.27–291.94 m^2^/g, of which the highest EAI was found at the highest pH studied (pH 8), and a strong positive correlation between solubility and emulsifying activity was found (Barac et al., 2010). The results of the current study were comparable to those of other studies in the literature. The pH‐dependent trend of protein solubility is similar to the EAI and ESI of MTP and MTY since many of their functional attributes rely on their ability to dissolve initially (Radha & Prakash, 2009). Lower EAI and ESI were probably due to increased protein–protein interactions and reduced solubility, which led to a decrease in flexibility and capability to form efficient interfacial membranes (Barac et al., 2010). EAI and ESI increased at pH below the isoelectric point since significant protein unfolding caused by the severe pH situation occurred and the ability of the proteins to form a stronger and viscoelastic network increased (Li et al., 2022).

#### 3.5.4 Foaming characteristics

FC of MTP and MTY varied from 35% to 55% and 35% to 86%, whereas FS of MTP and MTY ranged from 7.14% to 75.68% and 1.39% to 61.67%, respectively (Figure 1). The maximum FC and FS of both MTP and MTY were found at pH 11 and 7, whereas the lowest FC and FS were observed at pH 3–5. In accordance with our study, the FC and FS of Chinese quince seed protein isolate (CQSP) were minimum (22.5%) at pH 4. In addition, our finding was comparable to that of Fathollahy et al. (2021), who found that Persian lime seed protein isolate had the lowest FC and FS at pH 4, and had the highest FC and FS at pH 10–12 and 8, respectively. In another study, as the pH increased from 5 to 10, the FC increased at the same time (Deng et al., 2019). It was attributed to a lack of proteins available to diffuse at the water/air interface and form bubbles. The FC and FS of hemp seed protein isolate (95.15% protein) were 69% and 54% at pH 7, respectively (Liu et al., 2023). A protein must be fully solubilized in order to form and maintain a foam. The changes in FC and FS are associated with an increase in protein solubility and improved stabilization of air bubbles by proteins. The findings of FS were ascribed to the possibility that MTP and MTY may undergo partial unfolding under neutral pH conditions, leading to the formation of a molten globule characterized by a well‐balanced amphiphilicity and a structurally more flexible state. MTY is higher in FC, which might be a reason for the higher non‐protein constituents such as gums in MTY, as protein–polysaccharide hybrid polymers increase their ability to produce a viscoelastic layer at the bubble surface and avoid film rupture due to the protein–gum conjugation (Amid et al., 2013). The FS of proteins mixed with polysaccharides increases, mainly due to an increase in viscosity. However, in this study, the FS of MTP, which has a higher purity, was found to be higher than that of MTY at neutral pH in particular. This might be related to the lower protein solubility of MTP at these pH levels, thus leading to a lower protein:gum ratio. Xu et al. (2020) stated that protein component is the main dominant factor for FS, and FS decreased with decreasing protein component in the mixture.

#### 3.5.5 Zeta potential

The zeta potential is a critical physical parameter that describes protein surface charge. ZP of MTP altered between −15.7 at pH 4 and −27.4 at pH 8, whereas ZP of MTY ranged between −20.6 at pH 4 and −34.3 at pH 9. Above the isoelectric point (pH > 3), the zeta potential of chia seed proteins was found to be negative (Timilsena et al., 2016). Proteins possess a surplus of positively charged moieties (–NH^3+^) compared to negatively charged moieties (–COO–) below the isoelectric point (IP), resulting in a net positive charge. On the other hand, the amount of carboxyl outnumbers amino groups when the pH rises over IP, leading to the protein having a net negative charge. A similar trend with other studies regarding protein isolates was noticed in this study. The zeta potentials of eight varieties of mung bean protein isolate (MPI) were between −35 and −30 at pH 7 (Sun et al., 2022). Zeta potential of flaxseed protein isolate (FPI) was displayed in the pH range of 2.0–8.0, and the highest negative charge was noticed at pH of 8.0 due to enhanced anionic group exposure on the protein surface. As pH dropped, the absolute zeta potential of FPI declined to zero at an isoelectric pH of 4.2 (Kaushik et al., 2016). Zeta potentials of *Vicia faba* L. protein isolates (85.5%) were found at −20.86, −28.73, and −34.03 at pH 5.0, 6.8, and 8.0, respectively (Żmudziński et al., 2021). The zeta potential of ginkgo seed protein isolate was the highest at pH 10–11, while the lowest zeta potential was obtained at pH 2 (Zhang et al., 2021). However, no correlation was found between solubility and zeta potential. Changes in ionic strength can shift the pH at which a protein becomes electrically neutral, influencing its overall charge and, consequently, its zeta potential. The addition of polyphenols increased absolute zeta potential from −13.52 to around −26.50, indicating stronger interparticle electrostatic repulsion between protein molecules (Li et al., 2020). Polyphenols, which are a diverse group of naturally occurring compounds found in milk thistle seed, can interact with proteins and potentially lead to an increase in absolute zeta potential values (Choe et al., 2019).

#### 3.5.6 Antioxidant capacity

Antioxidative proteins increase the endogeneous antioxidant activity of foods, thus offering the use of potential antioxidant additives. In the DPPH assay, the IC50 of MTP was found to be 6.03 g/L, while the IC50 of MTY was 5.16 g/L. The IC50 value of protein extract from *Porphyra columbina* was 4.20 g/L, which is close to our values (Cian et al., 2012). The okra seed had an IC50 value of 6.38 g/L (Adetuyi & Ibrahim, 2014). Amaranth and tomato seed protein isolates showed low IC50 of 0.44 and 0.004 g/L, reflecting high antioxidant activity (Escudero et al., 2011). The IC50 of hemp seed protein hydrolysates ranged from 2.8 to 5.7 g/L (Tang et al., 2009). The IC50 of two chickpea protein isolates were found to be 167.4 and 130.2 g/L (Mesfin et al., 2021). The IC50 value of pure Spirulina protein concentrate for DPPH radical scavenging activities was found to be 8.08 g/L (Mosayebi et al., 2022). The higher and lower IC50 values were realized in the literature compared to MTP and MTY. Proteins display a wide range of antioxidant capacities depending on many factors, such as inactivating ROS, chelating prooxidative transition metals, reducing hydroperoxides, changing the physical characteristics of food systems, and scavenging free radicals (Elias et al., 2008). MTP and MTY did not differ from other proteins in a favorable manner in terms of antioxidant capacity. In the FRAP assay, the FRAP value of MTP was 166.3 μg GA/g protein, while the FRAP value of MTY was 180.0 μg GA/g protein. The FRAP activity of different soybean seeds (which contain >35% protein) was in the range of 0.19–1.58 mg of ascorbic acid equivalent (AAE) per g of dried seed weight (Choi et al., 2021). Palmaria palmata protein isolate had a FRAP value of 1.08 mg trolox equivalent per g dried protein sample (Harnedy et al., 2017). Li et al. (2014) found that the FRAP value of longan seed protein concentrate (59.8% protein) was 365.85 mg AAE/g concentrate. The FRAP assay also showed that the antioxidant capacity of MTP and MTY is lower compared to the other protein isolates/or concentrates, which may be derived from the presence of phenolics and various antioxidants in other proteins owing to impurity.

### 3.6 FTIR

**FIGURE 2:**
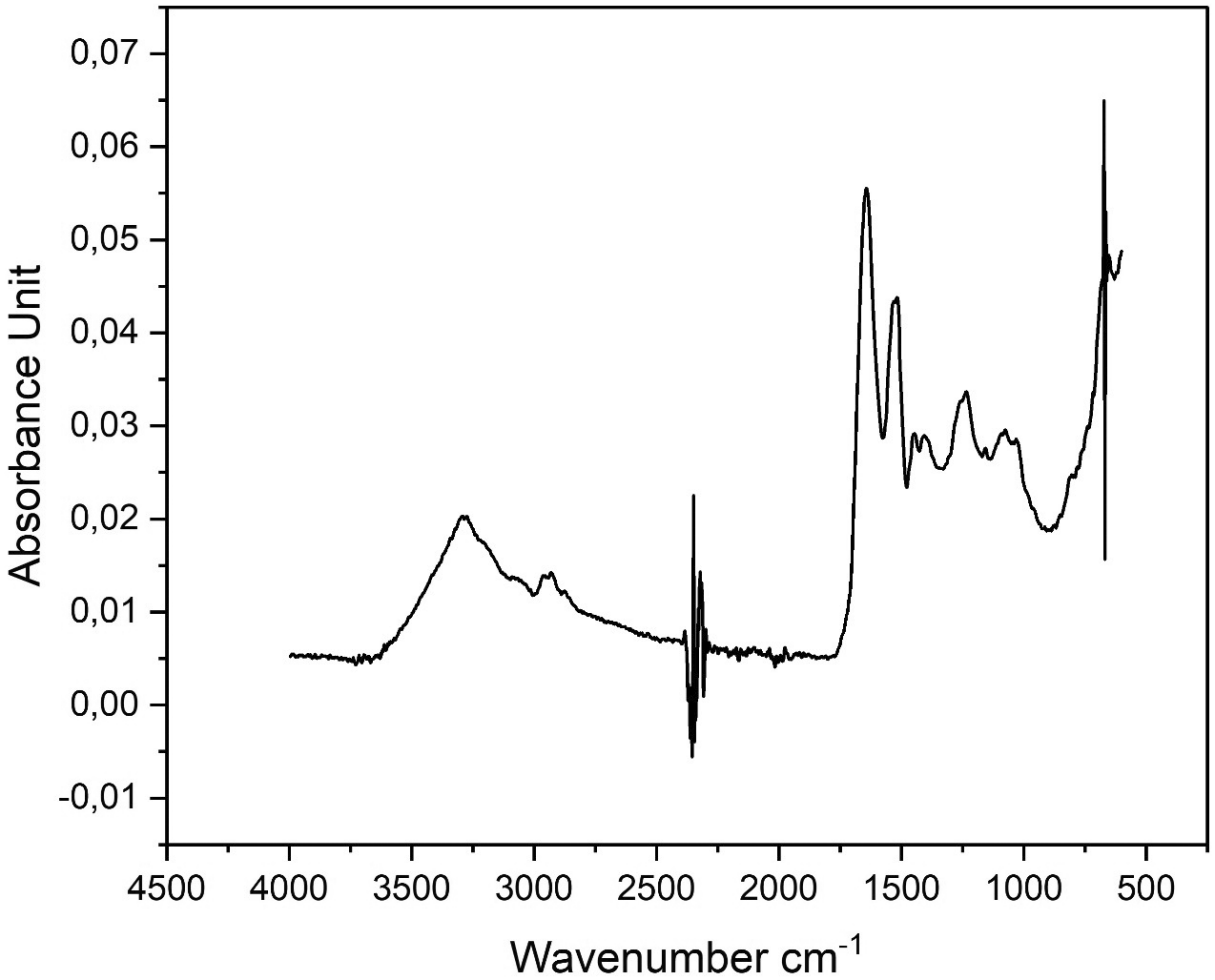
FTIR spectra of milk thistle protein with high purity (MTP).

The FTIR result of milk thistle protein with high purity (MTP) is illustrated in Figure 2. The peaks at 625–767 cm^−1^ denote Amide IV OCN bending. The peaks at 640–800 cm^−1^ imply Amid V out‐of‐plane NH bending. The peak at 1234 cm^−1^ indicates Amid III, N‐H bending/C‐N stretching. The 1400 cm^−1^ band is associated with free COO‐ groups. The bands between 1480 and 1575 cm^−1^ represent Amid II N‐H bending/C‐N stretching. The peaks at 1600–1690 cm^−1^ and the peak at 3300 cm^−1^ denote Amid I C=O stretch and Amide A N‐H bending, respectively (Aboul‐Enein et al., 2014).

**FIGURE 3:**
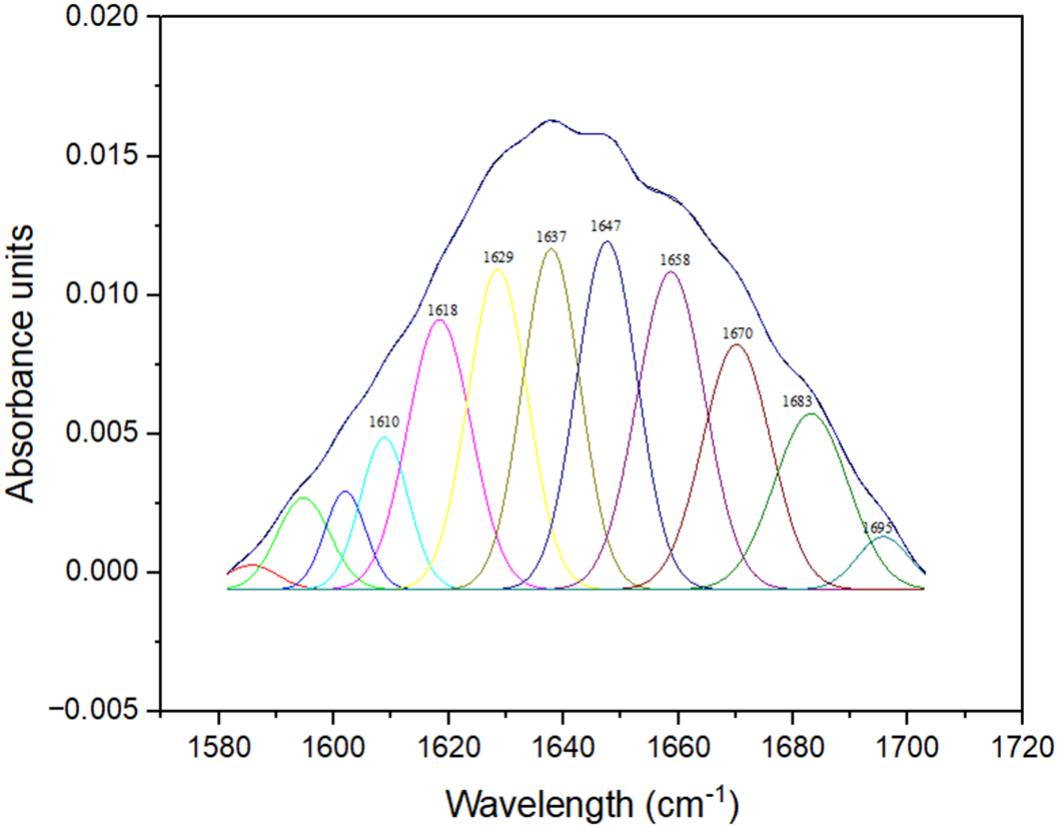
Deconvoluted FT‐IR spectra of the milk thistle protein with high purity (MTP).

The Amide I region is often used to characterize the secondary structure of proteins (Figure 3). The bands at 1610–1615 cm^−1^ are associated with the protein aggregates and/or side‐chain vibration. The bands at 1618–1620 cm^−1^ and 1680–1688 cm^−1^ represent anti‐parallel β‐sheets. The bands appearing around 1629–1633 cm^−1^ indicate the β‐strand structure. The bands around 1630–1638 cm^−1^ denote β‐sheet. The bands at 1643–1645 cm^−1^ and 1650–1660 cm^−1^ indicate random coil and α‐helix, respectively. The bands appeared at 1660–1680 cm^−1^ and 1690–1695 cm^−1^, respectively, implying β‐turns and β‐type structures (Shevkani et al., 2019).

The secondary structure content of milk thistle protein extract is given in Table 5. The secondary structure of the samples consisted of 2.25% β‐type, 12.06% β‐turns, 13.76% β‐strands, 14.18% β‐sheet, 15.23% random coil, 15.55% α‐helix, and 21.74% anti‐parallel β‐sheet. The secondary structure of milk thistle proteins was dominated by β‐sheets, β strands, and β‐turns, whereas α‐helix and random coil contents were in a relatively minor proportion (Table 5). Li et al. (2013) reported that milk thistle protein isolate extracted from *Slybum marianum* seed was composed of 0.3% α‐helix, 61.5% combined regular and distorted β‐strands, 11.8% β‐turns, and 26.4% random coil.

**TABLE 5:** Proportion of secondary structural components of milk thistle protein.

Observed wavelength (cm^−1^)	Proportion (%)	Assignment to secondary structure component
1610	5.23 ± 0.35	Side‐chain vibration
1618	12.35 ± 1.40	Anti‐parallel β‐sheet
1629	13.76 ± 1.20	β‐Strand
1637	14.18 ± 1.54	β‐Sheet
1647	15.23 ± 1.65	Random coil
1658	15.55 ± 2.36	α‐Helix
1670	12.06 ± 0.96	β‐Turns
1683	9.39 ± 0.60	Anti‐parallel β‐sheet
1695	2.25 ± 0.10	β‐Type

The difference in secondary structure may result from the isolation of different types of proteins, depending on the extraction conditions. A study conducted by Albe‐Slabi et al. (2022) revealed that the secondary structures of lupin proteins isolated using different pHs changed depending on the pH, and the alpha helix structure was better preserved at high pH (Albe‐Slabi et al., 2022). The secondary structures of proteins are closely related to their physical properties and biological activities (Liu et al., 2020). Pulse proteins with a greater proportion of β‐structures, including parallel and antiparallel β‐sheets and β‐turns, exhibited enhanced thermal stability and denatured at elevated temperatures compared to those with a higher α‐helix content (Carbonaro et al., 2012; Parmar et al., 2017). The prevalence of β‐structures in the secondary structure also contributes to protein digestibility. A high β‐sheet content has been reported to limit proteolytic enzyme access, resulting in lower protein digestibility (Yu, 2005). Due to their high β sheet content, milk thistle proteins have a very high possibility of showing high thermal stability and low digestibility.

### 3.7 SDS‐PAGE

**FIGURE 4:**
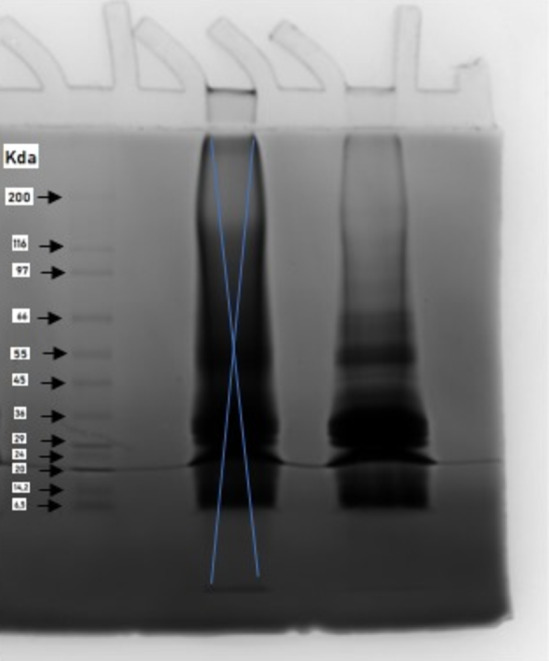
SDS‐PAGE patterns of the milk thistle protein with high purity (MTP).

SDS‐PAGE image of the extracted protein with high purity (MTP) is shown in Figure 4. The molecular weights of the extracted proteins varied between 116 and 6.5 kDa. The part where the bands are most intense was between 20 and 36 kDa. Also, there was a dense band formation around 45–55 kDa. Therefore, it could be deduced that most of the molecular weights of the extracted proteins were between 20 and 36 kDa. The results are consistent with the literature. Li et al. (2013) reported that the molecular weights of isolated milk thistle proteins varied between 16.3 and 112.1 kDa, and the strongest and broadest bands were resolved between 23.0 and 36.9 kDa.

## 4 CONCLUSION

In summary, we systematically optimized the extraction parameters for milk thistle seed proteins, considering both yield and purity, and subsequently characterized the extracted proteins. The optimal conditions for achieving maximum protein purity were determined to be a pH of 9.47, a temperature of 30°C, and an extraction time of 180 min. On the other hand, optimal conditions for maximizing protein yield were identified as a pH of 12, a temperature of 50°C, and an extraction time of 167 min. The oil absorption capacity, emulsifying properties, foaming capacity, and stability of both MTP and MTY were found to be comparable to those reported in various seed protein studies. The highest protein solubilities for both proteins were observed at pH 11, and protein solubility follows a pH‐dependent pattern that is similar to MTP and MTY's ESI and EAI. Additionally, at pH 4, both proteins exhibited zeta potential values closest to zero, indicative of their proximity to the isoelectric point. Despite lower antioxidant capacities compared to other protein isolates/concentrates, milk thistle proteins demonstrated significant potential for thermal stability and low digestibility, attributed to their elevated β‐sheet concentration. These findings highlight the multifaceted characteristics of milk thistle proteins, positioning them as promising candidates for diverse applications in the food and pharmaceutical industries.

## AUTHOR CONTRIBUTIONS

**Muhammed Ozgolet:** Data curation (equal); investigation (equal); writing – original draft (equal). **Fatih Bozkurt:** Conceptualization (equal); data curation (equal); writing – original draft (equal). **Salih Karasu:** Conceptualization (equal); supervision (equal); writing – review and editing (equal). **Osman Sagdic:** Methodology (equal). **Zeynep Hazal Tekin Cakmak:** Data curation (equal); investigation (equal).

## FUNDING INFORMATION

The authors express thanks to Türkiye Bilimsel ve Teknolojik Araştırma Kurumu (TUBITAK) for providing open‐access publication fee.

## CONFLICT OF INTEREST STATEMENT

The authors declare no conflict of interest.

## ETHICS STATEMENT

The manuscript has no ethical issue.

## CONSENT FOR PUBLICATION

All authors agreed to the publication of this manuscript.

## Data Availability

The data that support the findings of this study are available on request from the corresponding author.
