# Correction to “Optimization of extraction parameters of protein isolate from milk thistle seed: Physicochemical and functional characteristics”

**DOI:** 10.1002/fsn3.4457

**Published:** 2024-10-15

Ozgolet, M., Cakmak, Z. H. T., Bozkurt, F., Sagdic, O., & Karasu, S. (2024). Optimization of extraction parameters of protein isolate from milk thistle seed: Physicochemical and functional characteristics. *Food Science & Nutrition*, 12, 3346–3359. https://doi.org/10.1002/fsn3.4001

In the Acknowledgements section, the authors erroneously omitted that this study had been produced from a PhD thesis. Therefore the sentence, “The authors would like to acknowledge that this paper is submitted in partial fulfillment of the requirements for the PhD degree at Yildiz Technical University,” should replace the present sentence: “The authors express thanks to Yildiz Technical University for providing financial support for the research project.”

We apologize for this error.

